# Leaf Bacteriome in Sugar Beet Shows Differential Response against *Beet curly top virus* during Resistant and Susceptible Interactions

**DOI:** 10.3390/ijms23158073

**Published:** 2022-07-22

**Authors:** Rajtilak Majumdar, Carl A. Strausbaugh, Eric D. Vincill, Imad Eujayl, Paul J. Galewski

**Affiliations:** Northwest Irrigation and Soils Research, United States Department of Agriculture (USDA)—Agricultural Research Service (ARS), Kimberly, ID 83341, USA; carl.strausbaugh@usda.gov (C.A.S.); eric.vincill@usda.gov (E.D.V.); imad.eujayl@usda.gov (I.E.); paul.galewski@usda.gov (P.J.G.)

**Keywords:** sugar beet, BCTV resistance, beet curly top, microbiome, bacterial marker, virus resistance, leaf bacteriome, *Beta vulgaris*

## Abstract

*Beet curly top virus* (BCTV) significantly reduces sugar beet yield in semi-arid production areas. Genetic resistance to BCTV is limited; therefore, identification of additional resistance-associated factors is highly desired. Using 16S rRNA sequencing and BCTV resistant (R) genotypes (KDH13, KDH4-9) along with a susceptible (S) genotype (KDH19-17), we investigated leaf bacteriome changes during BCTV post inoculation (pi). At day 6 (~6-week-old plants), *Cyanobacteria* were predominant (~90%); whereas, at week 4 (~10-week-old plants) *Firmicutes* (11–66%), *Bacteroidetes* (17–26%), and *Verrucomicrobia* (12–29%) were predominant phyla and genotype dependent. Both *Bacteroidetes* and *Verrucomicrobia*, increased post infection only in the R lines. The bacterial genera *Brevibacillus* increased at 6 dpi, and *Akkermansia* and *Bacteroides* at 4 wkpi in the R lines. Linear discriminant analysis effect size (LEfSe) identified potential biomarkers in the R vs. S lines. Functional profiling revealed bacterial enrichment associated with the TCA cycle, polyisoprenoid, and L-methionine biosynthesis pathways only in KDH4-9 at 6 dpi. At 4 wkpi, bacteria associated with tryptophan and palmitate biosynthesis in the R lines, and uridine monophosphate, phosphatidyl glycerol, and phospholipid biosynthesis in the S line, were enriched. Future characterization of bacterial genera with antiviral properties will help establish their use as biocontrol agents/biomarkers against BCTV.

## 1. Introduction

Sugar beet (*Beta vulgaris* L.) is an economically important crop in the mid and western parts of the United States (U.S.). This crop contributes to the production of ~55% of the total sugar produced in the U.S. Among the different pests and pathogens that significantly reduce yield and sugar production in sugar beet, *beet curly top virus* (BCTV) is a major concern in the semi-arid areas of the U.S. [1,2]. The virus also infects other commercially important crops, such as tomatoes, peppers, spinach, common beans, cucurbits, coriander, and hemp [3,4,5,6,7,8]. This single-stranded DNA virus, belonging to the genus *Curtovirus*, is vectored by the beet leafhopper (BLH; *Circulifer tenellus* (Baker)) which prefers feeding on the leaves of sugar beet plants and transmits BCTV. Severe outbreaks of this disease can cause a yield reduction by more than 30% [9]. Genetic resistance to BCTV in sugar beet is low to intermediate in commercial cultivars [2,7,10]. Thus, host resistance in commercial sugar beet production has to be supplemented by neonicotinoid seed treatments that control the BLH vector. However, the use of neonicotinoid insecticides may be restricted in the future because of environmental concerns [11,12]. So, there is an urgent need to explore other factors that improve host plant resistance against BCTV in a more environmentally friendly manner. The microbiome, in this regard, can be an important factor as they have been shown to play critical roles in host plant resistance against diverse pests and pathogens [13,14]. The microbiome contributes to resistance by modulating host metabolic pathways associated with defense responses, through the production of metabolites that possess direct antimicrobial properties or could simply outcompete pathogens by competing for common resources in plant tissues without being detrimental to the plants.

In nature, co-evolution of plants and their associated microbiomes plays an important role in plant adaptation to changing environments, conferring tolerance against abiotic and biotic stress, as reviewed in [13,15]. Among the different taxonomical groups that constitute host microbiome, bacteria are the most abundant microbial community and have been well studied in both plants and animals [16]. The microbiome is not only important in maintaining optimum plant health, but it has also been shown to alleviate disease symptoms during viral infections [16,17]. A protein elicitor, PeBA1, produced by *Bacillus amyloliquefaciens* NC6 strain-induced systemic resistance against *tobacco mosaic virus* (TMV) and against the fungal pathogen *Botrytis cinerea* Pers. in *Nicotiana benthamiana* Domin and *Nicotiana tabacum* L. This systemic resistance was attributed to induced expression of defense-related genes involved in the production of jasmonic acid, salicylic acid, and phenylalanine ammonia lyase etc., by the PeBA1 elicitor protein [18]. In tomato (*Solanum lycopersicum* L.), higher abundance of bacterial genera such as *Actinobacteria* sp., *Pseudomonas* sp., and *Agrobacterium* sp. in the rhizosphere soil was associated with reduced *tomato spotted wilt virus* (TSWV) infection and reduced disease symptoms [19]. The role of the microbiome in plant resistance against viruses depends upon several factors including host plant microbiome composition/diversity, developmental stage, genetic background, and the involvement of defense-related signaling pathways. These factors can play critical roles in restricting viral replication, transmission/movement of the virus, and development of disease severity in host plants. Unravelling the pattern of the microbiome assembly in disease-resistant germplasms under natural conditions and restructuring of microbiome composition during disease conditions will improve our understanding towards the development of novel mitigation strategies. Manipulating the host plant microbiome to improve host plant resistance and augment agricultural production will be key towards achieving an eco-friendly approach in the future.

As BLH feeds on the phloem of sugar beet plants, BCTV is transmitted and any leaf-associated factors such as leaf bacteriome contributing to host plant resistance against the virus will be of great importance towards the development of resistant germplasm and plant protection strategies. The role of the leaf microbiome against foliar pests and pathogens have been reported in earlier studies [20]. In general, scientific data delineating any potential role of the host plant microbiome in resistance against viruses are rather limited when compared to fungal and bacterial pathogens. Microbiome-related work in sugar beet has primarily focused on the potential role of the microbiome, mainly against fungal pathogens by comparing the rhizosphere microbiome of *Beta vulgaris* ssp. maritima (beet crop ancestor) and modern sugar beets, *Beta vulgaris* [21], field performance and disease resistance [22], genotypic differences in postharvest storage quality and disease resistance [23] etc. How the microbiome may contribute to sugar beet resistance against viral pathogens is poorly understood. No information is available on how the leaf microbiome in sugar beets responds to BCTV infection during the early stages when disease symptoms are not evident. This might have positive consequences on host plant resistance and development of diseases symptoms during the later stages of infection. This is the first report on sugar beet leaf bacteriome response against BCTV. Using BCTV-susceptible (S; KDH19-17: Line 19) and BCTV-resistant (R; KDH13: Line 13 and KDH4-9: Line 4) sugar beet genotypes and BLH-mediated natural BCTV infection, we investigated changes in the leaf bacterial microbiome at early (6 d post inoculation; 6 dpi) and late (4 weeks post inoculation; 4 wkpi) infection stages. Data presented here highlight restructuring of bacterial microbiome in the leaves following BCTV infection and their differential regulation in the R vs. S sugar beet genotypes in a temporal manner. This work also identifies potential bacterial biomarkers in the leaves of R genotypes that might have an implication in BCTV resistance.

## 2. Results

Post quality control, ~83,000 raw tags/sample and ~68,000 valid tags/sample mapping to zero-radius operational taxonomic units (zOTUs) were obtained (Table S1). Two different time points, early (6 dpi; no visual symptoms) and late (4 wkpi; visual leaf symptoms), were selected to study leaf bacteriome responses against BCTV along with uninfected control treatments and using BCTV-resistant and BCTV-susceptible sugar beet genotypes. Our microbiome sequencing was unable to resolve any fungal diversity in the leaf samples of the R and S sugar beet lines, possibly due to a lower relative abundance of fungal communities in the leaves in the developmental stages and growth conditions under the greenhouse environment used in this study. As the bacterial microbiome was predominant in the leaves, we therefore focused on understanding any changes in leaf bacteria in relation to BCTV resistance and/or susceptibility.

### 2.1. Leaf Bacterial Phyla and Genera Showed Significant Changes between the Resistant and Susceptible Lines with BCTV Infection

**Figure 1:**
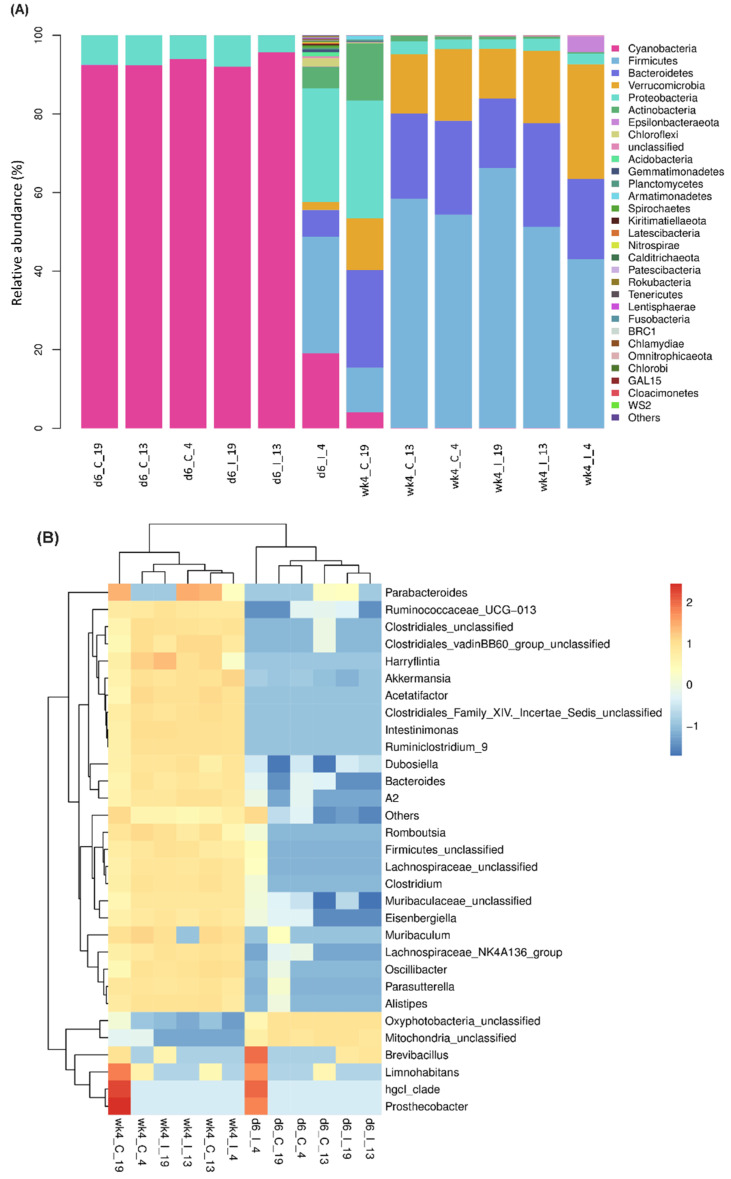
Bacterial phyla and genera were significantly altered among *beet curly top virus* (BCTV)-susceptible and BCTV-resistant sugar beet genotypes. Mean relative abundance of (**A**) prokaryote phyla; and (**B**) genera in the leaves at early (day 6; d6) and late (week 4; wk4) time points with or without BCTV infection. 19, susceptible genotype; 13 and 4, resistant genotypes. C = uninfected control, and I = BCTV-infected. The data are mean ± standard error of four replicates (uninfected control) and five replicates (infected).

Overall, a total of 49 different bacterial phyla were detected in the leaves of the R and S lines across all samples, with only 23 phyla showing a minimum abundance of 0.1% and/or higher (Figure 1A; Table S2). At 6 d (~6-week-old plants), in uninfected leaves of both R and S lines, bacteria belonging to the phylum *Cyanobacteria* were predominant (~92%) and were followed by *Proteobacteria* (6–7%). Other bacterial phyla whose relative abundance varied between 0.01 and 0.09% included *Bacteroidetes*, *Firmicutes*, and *Verrucomicrobia*. *Actinobacteria* content were lower (0.01) in the R lines in comparison to the S line (0.02%). No significant differences in other bacterial phyla were observed between the R and S lines. The resistant Line 4 showed the most positive changes in several bacterial phyla following BCTV infection at 6 dpi. Examples of phyla whose relative abundance were ≥5% at 6 dpi in Line 4 include *Firmicutes* (30%), *Proteobacteria* (29%), *Bacteroidetes* (7%), and *Actinobacteria* (5%). A greater change in the relative abundance of bacterial phyla was observed at 4 weeks. In the uninfected leaves at 4 weeks (~10-week-old plants), bacteria belonging to *Firmicutes* were highest and R lines showed a higher abundance (54–58%) than the S line (11%). Other bacterial phyla with >12% in the R and S lines included *Bacteroidetes* (23–24%), *Verrucomicrobia* (13–18%) etc. *Proteobacteria* (2–3%) and *Actinobacteria* (~1%) were significantly lower in the R lines in comparison to the S line (*Proteobacteria*: 30%; *Actinobacteria*: 15%). At 4 wkpi, *Firmicutes* were higher (66%) only in the S line (vs. control; 11%). Both *Bacteroidetes* (20–26%) and *Verrucomicrobia* (18–29%) were higher in the R lines in comparison to the S line (*Bacteroidetes*: 18%; *Verrucomicrobia*: 13%) at 4 wkpi. Bacteria belonging to the phylum *Epsilonbacteraeota* were higher (4%) only in the resistant Line 4 at 4 wkpi in comparison to the other two lines (>0.01%).

Among different bacterial genera (Figure 1B; Table S3), *Oxyphotobacteria* comprised 92–94% of the bacterial community followed by *Mitochondria* (5–8%) in the uninfected leaves of control plants of the R and S lines at 6 d. *Brevibacillus* was highly abundant (28%) at 6 dpi, mainly in the resistant Line 4 compared to the susceptible Line 19 (0.03) and the other resistant line 13 (0.04). Similarly, *Limnohabitans* showed a higher abundance (3%) only in the resistant line 4 at 6 dpi, in comparison to the other lines where it was almost undetectable. Viral infection reduced the relative abundance of *Mitochondria* in both R lines (2–4%) in comparison to the S line (8%). The relative abundance of bacterial genera varied more among the samples at 4 weeks. In uninfected leaves of control plants, the relative abundance of *Akkermansia* was highest (up to 18%), followed by *Muribaculaceae* and *Bacteroides* (up to 10%). The resistant lines showed a higher abundance of these bacterial genera and including other genera, *Acetatifactor* and *Clostridiales*, in comparison to the susceptible line. Examples of bacterial genera whose abundance were up to 3–8% in the uninfected leaves included *Ruminococcaceae*_UCG-013, *Lachnospiraceae*_NK4A136_group, *Lachnospiraceae*, *Parabacteroides* etc. Upon infection, *Akkermansia* and *Bacteroides* were higher in the R lines (vs. S line) at 4 wkpi; whereas, *Oxyphotobacteria* (0.02–0.03%) and *Ruminococcaceae*_UCG-013 (7–8%) were lower in the BCTV-infected leaves of R lines when compared to the S line (*Oxyphotobacteria*: 0.06% and *Ruminococcaceae*_UCG-013: 10%) at 4 wkpi.

### 2.2. Leaf Bacterial Diversity Was Significantly Altered following BCTV Infection in the Resistant vs. Susceptible Lines

#### 2.2.1. Alpha Diversity of Samples

**Figure 2:**
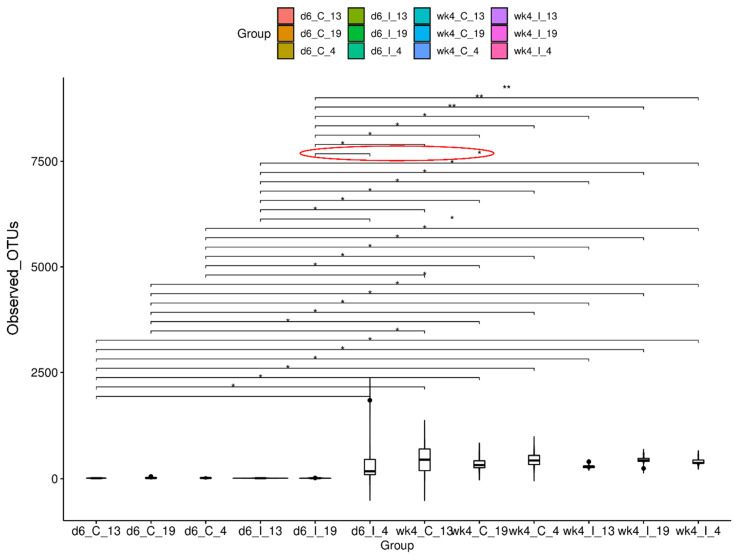
Alpha diversity of *beet curly top virus* (BCTV)-susceptible (19) and BCTV-resistant (13, 4) sugar beet genotypes with and without BCTV infection at early (day 6; d6) and late (week 4; wk4) time points. Observed OTU boxplot and Kruskal–Wallis pairwise comparisons (* *p <* 0.05, ** *p <* 0.01). C = uninfected control, and I = BCTV-infected. The significant difference between d6_I_4 and d6_I_19 (as mentioned in the text) is shown through red-lined ellipse. The data are mean ± standard error of four replicates (uninfected control) and five replicates (infected).

Alpha diversity measurement was used to estimate microbial diversity within a given sample and/or between treatment groups. Statistical comparisons of observed OTUs between sample groups were performed using Kruskal–Wallis pairwise comparisons (Figure 2). Among different comparisons within the control treatment among genotypes at a specific time point (day 6, d6; week 4, wk4) or within viral infection among genotypes at a specific time point or control vs. inoculated within a genotype at a specific time point, microbial diversity was significantly higher (*p* = 0.03) only in the infected leaves of Line 4 (d6_I_4; R) vs. Line 4 (d6_I_19; S) at 6 dpi. Detailed comparisons between all other treatment groups with any significant differences have been shown in Figure 2.

#### 2.2.2. Beta Diversity of Samples

**Figure 3:**
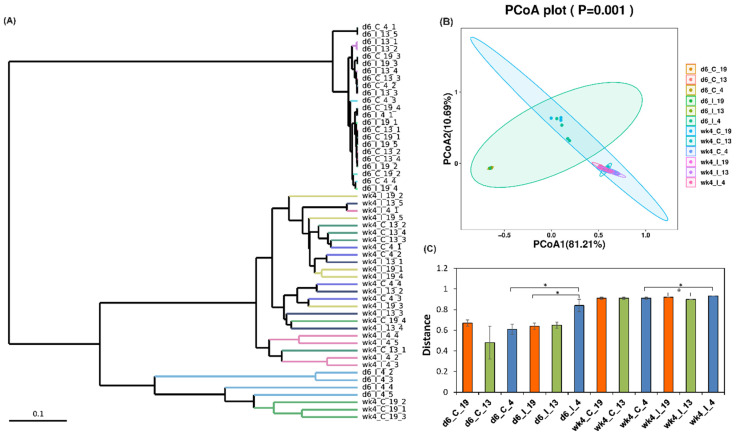
Beta diversity of *beet curly top virus* (BCTV)−susceptible and BCTV−resistant sugar beet genotypes with and without BCTV infection at early (day 6; d6) and late (week 4; wk4) time points. (**A**) Cluster dendrogram of different treatments, (**B**) principal coordinate analysis (PCoA), and (**C**) comparison of weighted UniFrac distances within and between control (**C**) and BCTV−infected (I) sugar beet susceptible (19) and resistant (13, 4) genotypes at early and late time points (* *p* < 0.05). C = uninfected control, and I = BCTV-infected. The data are mean ± standard error of four replicates (uninfected control) and five replicates (infected).

Beta diversity measurement was used to estimate microbial communities considering their relative abundance in a specific sample and their phylogenetic relationship. To compare microbial communities among samples, cluster dendrogram and principal coordinate analysis (PCoA) were used to visualize the data. UniFrac distance matrices were used to analyze microbial community structure and significant differences between microbial communities were determined through pairwise analysis of similarities (ANOSIM). The cluster dendrogram shows a hierarchical relationship between different treatments (Figure 3A). A high PCoA variation observed between PC1 (81.21%) and PC2 (10.69%) suggests a wide separation among treatments (Figure 3B). The infected or the uninfected samples of the resistant or susceptible lines were relatively clustered together and there was a spatial separation of samples collected at 6 d and 4 weeks. Comparison of weighted UniFrac distances between samples showed significant differences (*p* < 0.05) in the leaf bacterial community between infected Line 4 (d6_I_4; R) and Line 19 (d6_I_19; S) at 6 dpi, and between control (d6_C_4) and infected (d6_I_4) samples of Line 4 (R) at 6 d (Figure 3C). At 4 weeks, significant differences (*p* < 0.05) in the bacterial community were observed between wk4_I_19 and wk4_I_13, and between wk4_C_4 and wk4_I_4.

**Figure 4:**
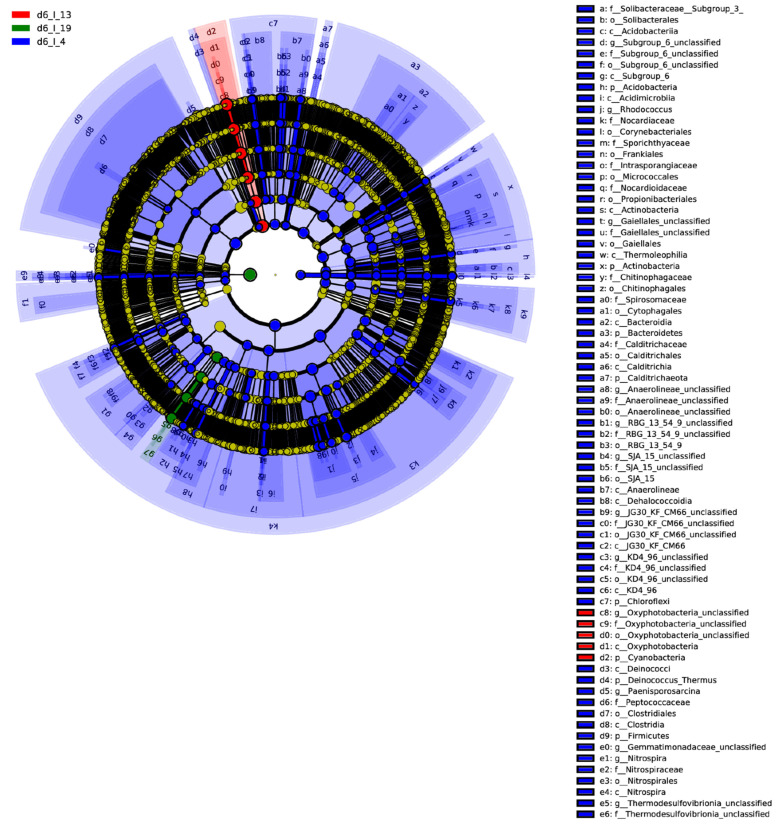
Linear discriminant analysis effect size (LEfSe) analysis of sugar beet genotypes at 6 days (d6) post inoculation. Hierarchal taxonomic cladogram displaying potential biomarkers of *beet curly top virus* in the susceptible (S) and resistant (R) sugar beet genotypes. 19, S genotype; 13 and 4, R genotypes; I = BCTV-infected.

**Figure 5:**
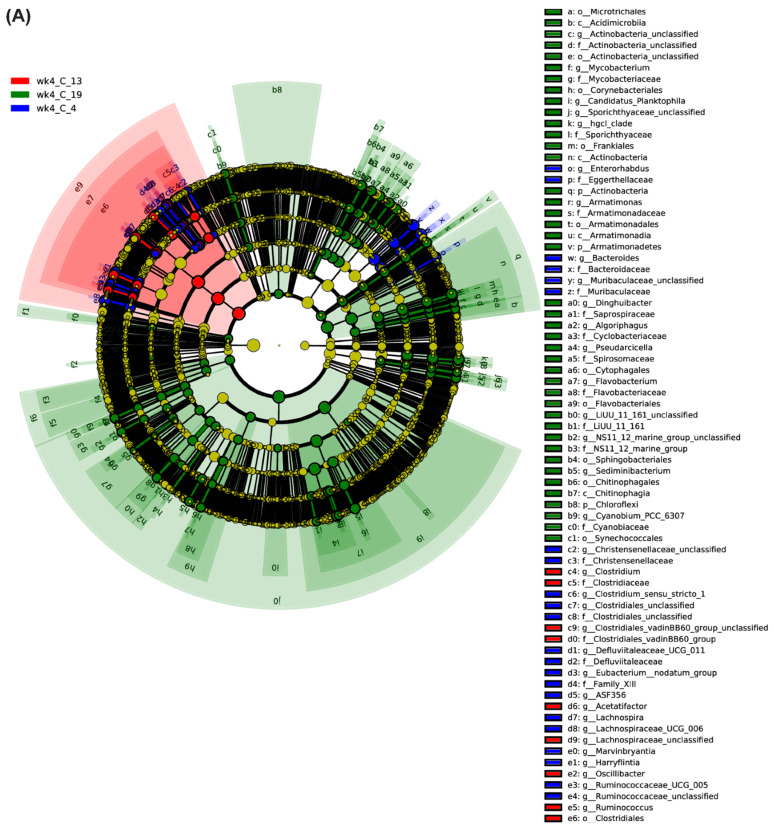

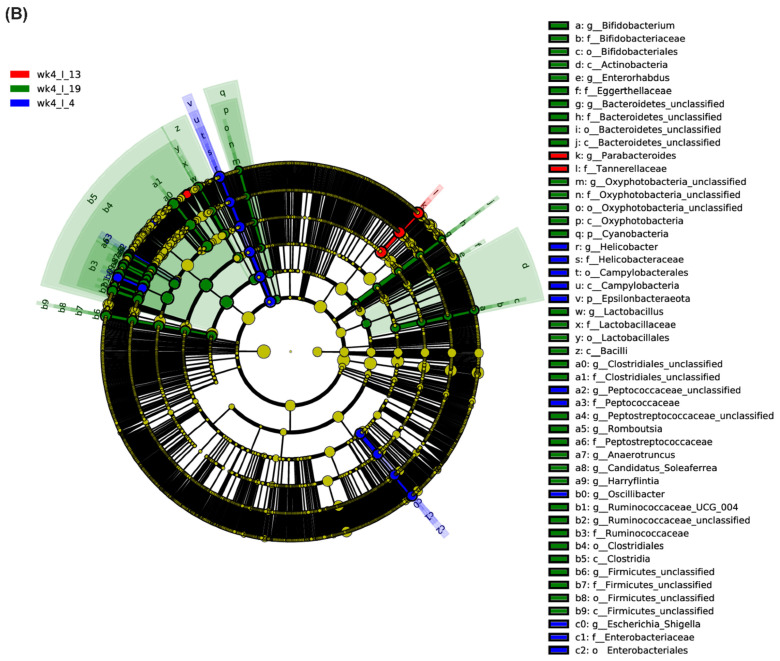
Linear discriminant analysis effect size (LEfSe) analysis of sugar beet genotypes at 4 weeks (wk4). Hierarchal taxonomic cladogram displaying potential biomarkers of *beet curly top virus* in the (**A**) control, and (**B**) BCTV-infected susceptible and resistant sugar beet genotypes. 19, susceptible genotype; 13 and 4, resistant genotypes; C = uninfected control, and I = BCTV-infected.

Linear discriminant analysis effect size (LEfSe) analysis was performed to identify any potential biomarkers linked to BCTV resistance in the BCT-resistant sugar beet genotypes used in this study. At day 6 (Figure 4 and Figure S1), examples of bacterial phyla that increased (*p* < 0.05) and exhibited an LDA score >4.5–5.0 in the virus-infected samples include *Oxyphotobacteria*, *Cyanobacteria* (Line 19; S); *Mitochondria*, *Rickettsiales* (Line 13; R); and *Firmicutes*, *Proteobacteria*, *Bacteroidetes* (Line 4; R). At week 4, examples of bacterial phyla associated with uninfected control samples (Figure 5A and Figure S2A) included *Clostridia*, *Firmicutes* (Line 19; S); *Proteobacteria*, *Betaproteobacteriales* (Line 13; R); and *Bacteroides*, *Muribaculaceae* (Line 4; R). Examples of bacterial phyla associated with BCTV-infected samples at week 4 (Figure 5B and Figure S2B) included *Tannerellaceae*, *Parabacteroides* (Line 19; S); *Clostridia*, *Ruminococcaceae* (Line 13; R); and *Helicobacteraceae*, *Campylobacterales* (Line 14; R).

### 2.3. Predicted KEGG Pathways Were Differentially Regulated in the Resistant vs. Susceptible Lines

To gain further insights into relationships between presence/absence and/or increase/decrease in specific bacteria in the leaves of sugar beet genotypes in response to BCTV infection and their potential biological roles in BCTV resistance, we performed functional profiling (KEGG pathway association) of bacterial communities.

**Figure 6:**
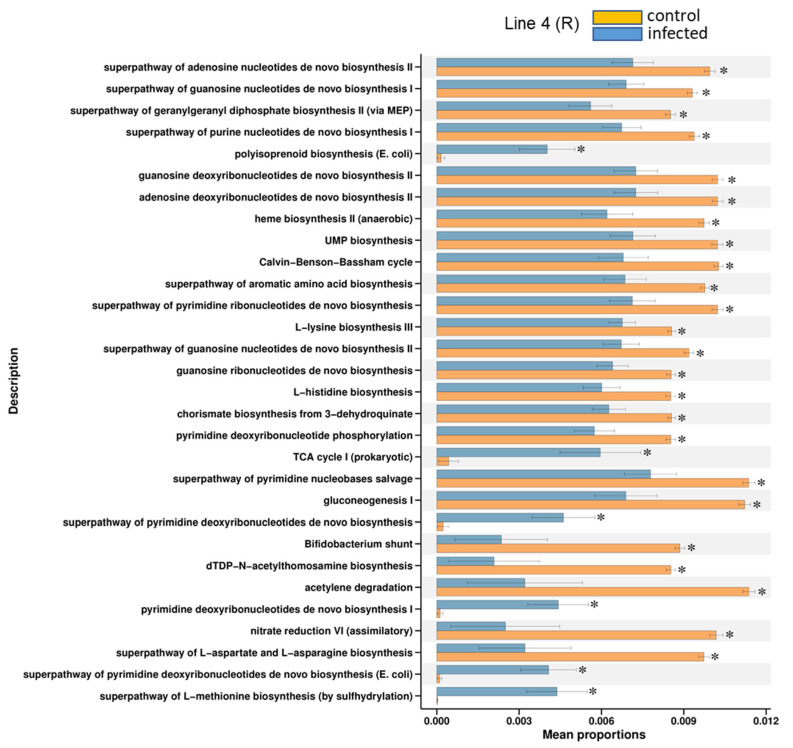
KEGG modules associated with leaf bacteria that were significantly different (* *p* < 0.05) between uninfected control (C) and *beet curly top virus* (BCTV)-infected (I) resistant sugar beet genotype, Line 4, at 6 days post inoculation (6 dpi). Data are mean ± standard error of four replicates (uninfected control) and five replicates (infected).

**Figure 7:**
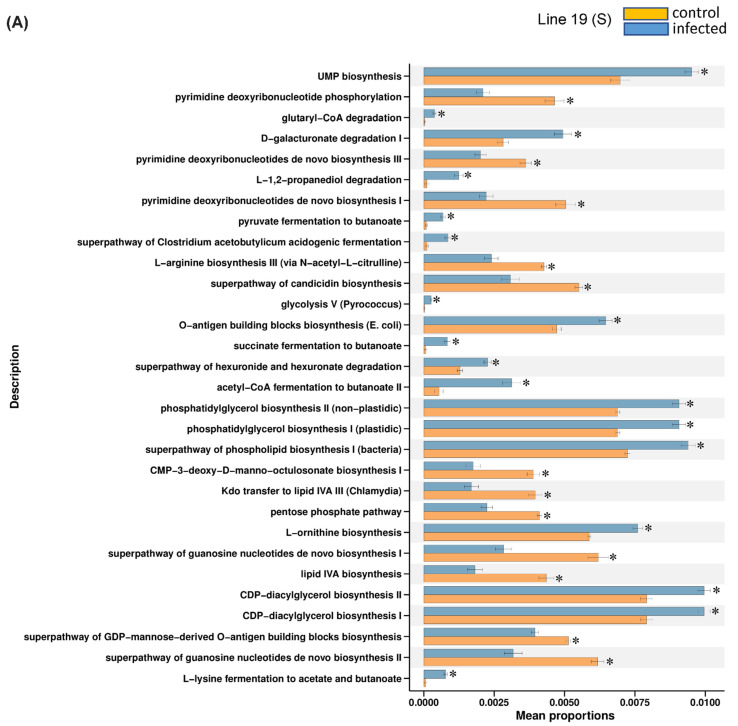

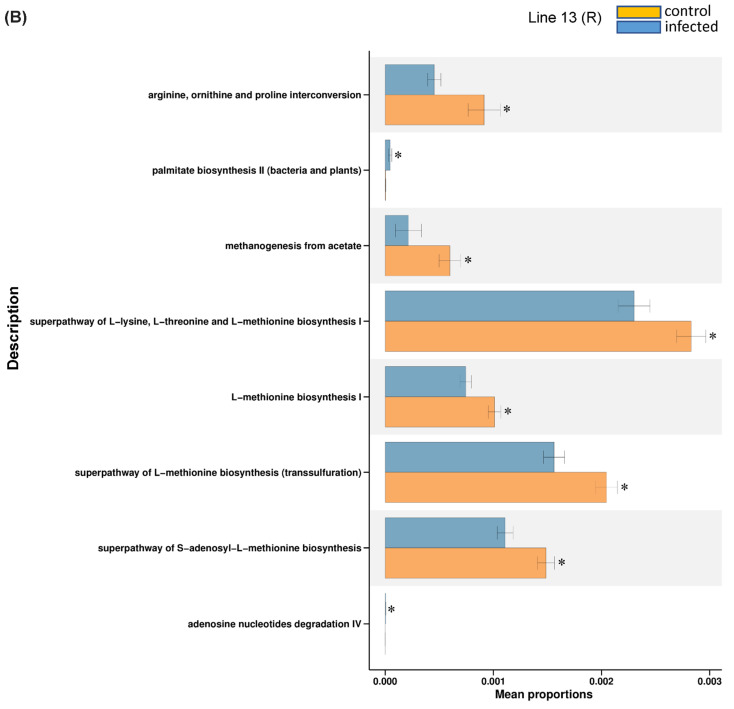

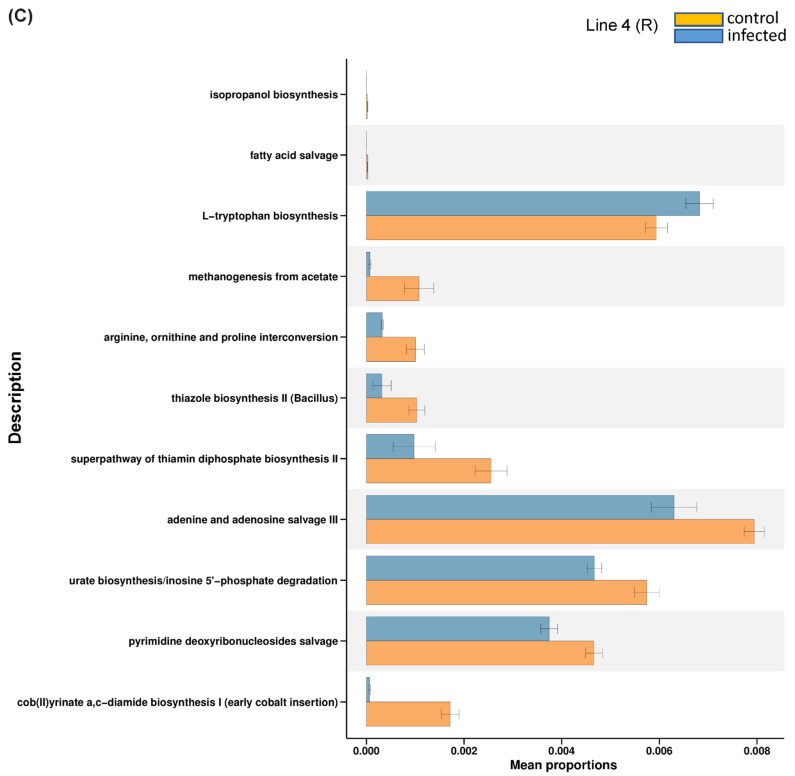
KEGG modules associated with leaf bacteria that were significantly different (* *p* < 0.05) between uninfected control (**C**) and *beet curly top virus* (BCTV)-infected sugar beet genotypes (**A**) Line 19 (susceptible; S); (**B**) Line 13 (resistant; R); and (**C**) Line 4 (resistant; R) at 4 weeks post inoculation (wkpi). Data are mean ± standard error of four replicates (uninfected control) and five replicates (infected).

In general, the majority of the KEGG pathways detected were mostly down-regulated (*p* < 0.05) in the BCTV-infected samples of Line 4 at day 6 (d6_I_4). Examples of KEGG pathways that were highly down-regulated in the infected samples of Line 4 included the super pathway of pyrimidine nucleobases salvage, gluconeogenesis, super pathway of L-aspartate and L-asparagine etc. (Figure 6). On the other hand, BCTV infection up-regulated the relative abundance of bacteria primarily associated with pathways such as polyisoprenoid biosynthesis, the super pathway of L-methionine biosynthesis (by sulfhydrylation), and the super pathway of pyrimidine deoxyribonucleotides de novo biosynthesis (Figure 6). No pathway enrichment of bacterial communities could be achieved for other treatments at day 6. At 4 wkpi, when disease symptoms were visible, examples of bacterial communities that were highly enriched and up-regulated in the infected samples of Line 19 (S) included pathways associated with CDP diacylglycerol biosynthesis I and II, phosphatidylglycerol biosynthesis I (plastidic) and II (non-plastidic), UMP biosynthesis etc. (Figure 7A). Higher enrichment of bacterial communities representing pathways such as the super pathway of L-lysine, L-threonine, and L-methionine were observed in Line 13 (R), though the control samples were higher than the infected samples (Figure 7B). Representation of bacteria associated with L-tryptophan biosynthesis were increased (Figure 7C) in the infected samples (vs. control) of Line 4 (R). Other bacterial communities associated with pathways such as adenine and adenosine salvage III, urate biosynthesis/inosine 5′-phosphate degradation were higher in the control samples of Line 4 at 4 wkpi (Figure 7C).

### 2.4. Correlation between Bacterial Communities across Treatments

**Figure 8:**
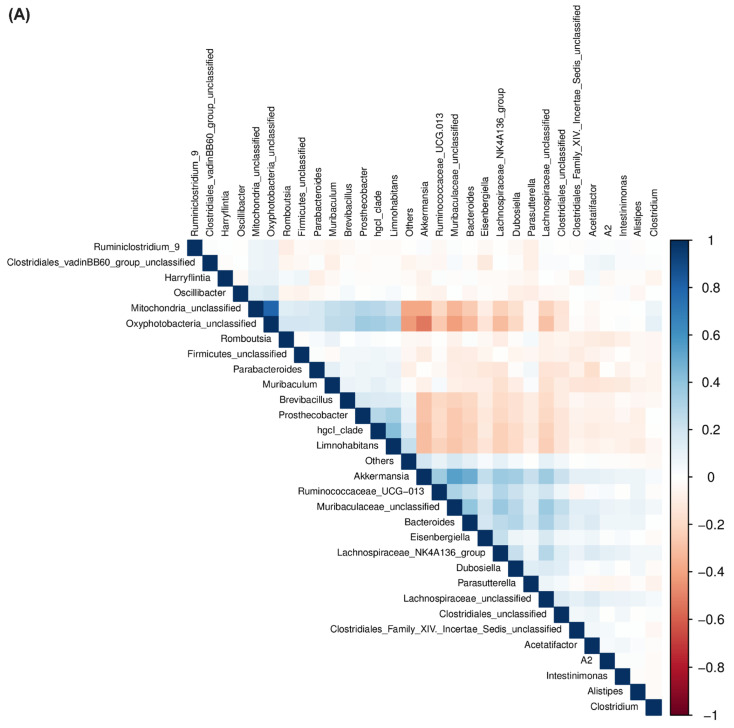

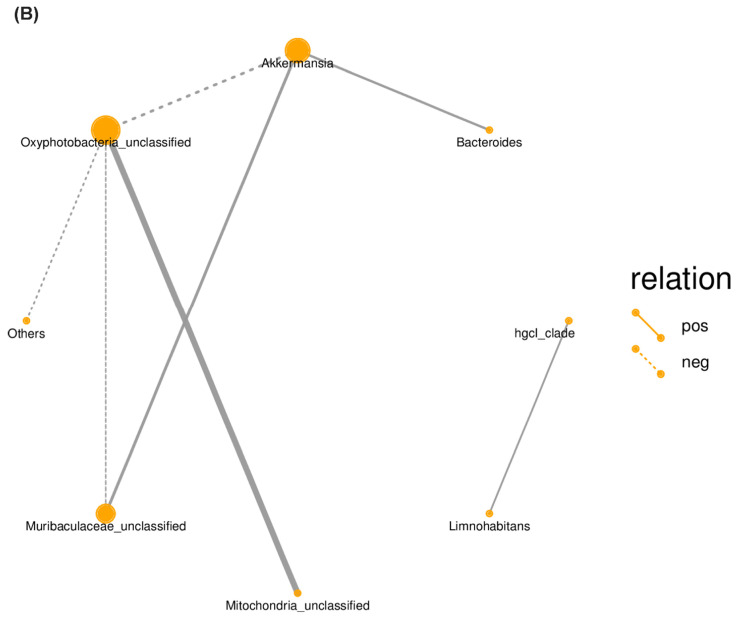
Sparse correlations for compositional data (SparCC). (**A**) correlation heatmap between different bacterial communities; (**B**) correlation network among bacterial communities. A solid line between two bacterial communities indicates a positive correlation, and a dotted line indicates a negative correlation between them. The thicker the solid line, the higher the value of positive correlation between them. The size of the nodes indicates the abundance of bacterial communities. The lower the abundance, the smaller the node.

To understand the relationships among bacterial communities and the co-occurrence of specific bacterial communities in the R and S lines with or without BCTV infection, a correlation matrix (Figure 8A), along with a correlation network (Figure 8B), were constructed including all treatments. A strong positive correlation (+0.80) was observed between *Oxyphotobacteria* and *Mitochondria*; whereas, a moderate positive correlation [(+)0.40–(+)0.55] was observed between *Akkermansia* and *Muribaculaceae*/*Bacteroides*, and hgcl_clade and *Limnohabitans*. A moderate negative correlation (−0.52) was observed between *Oxyphotobacteria* and *Akkermansia*, and between *Oxyphotobacteria* and *Muribaculaceae* (−0.42).

### 2.5. Susceptible Lines Exhibited Higher BCTV-Related Symptoms vs. Resistant Lines

**Figure 9:**
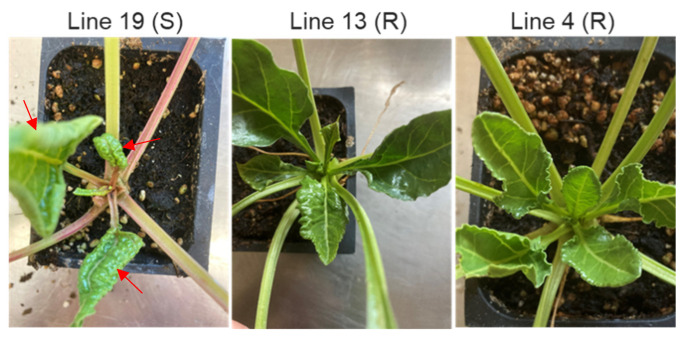
Disease symptoms in sugar beet plants that were previously infected by *beet curly top virus* (BCTV). Disease symptoms (leaf curling and swelling) in the apical leaves (indicated by red arrows) of *beet curly top virus* (BCTV)-susceptible (S; Line 19) and BCTV-resistant (R; Line 13 and Line 4) genotypes at 4 weeks post inoculation (wkpi).

Control and BCTV-infected plants of both S and R lines were evaluated for disease symptoms at 4 wkpi. The apical leaves of the S line (19) showed strong BCTV-associated disease symptoms such as leaf curling and thickening, and vein swelling (Figure 9). The R lines showed minimal to no disease symptoms. The uninfected control plants, that were kept in separate cages without any exposure to the viruliferous BLHs during the experiment, did not show any BCTV-associated disease symptoms and, hence, they are not shown here. Viral load estimation through RT-PCR showed a higher relative signal strength of BCTV capsid protein gene, *V1*, expression in the S line (vs. R lines) both at early 6 dpi [24] and late (4 wkpi) infection stages (Figure S3). The relative signal strength of *V1* gene expression in the S line (vs. R lines) was much higher at the late (4 wkpi) infection stage.

## 3. Discussion

The beneficial role of the host plant tissue-specific microbiome against pathogens have been explored in diverse plant species [13,15]. The leaf microbiome plays an important role in resistance against pathogens that are leaf-associated [14,20]. How the leaf bacteriome is affected by BCTV infection in sugar beet is unknown. As BCTV is transmitted through BLH during feeding on sugar beet phloem tissue, we therefore investigated any potential signatures of the leaf bacteriome associated with resistance against BCTV using virus-resistant sugar beet genotypes.

Developmental stages had a significant effect on the relative abundance of leaf bacteria in the sugar beet genotypes used in this study. There was a major shift in bacterial phyla in the leaves from 6 d (~6-week-old plants) to 4 weeks (~10-week-old plants), where *Cyanobacteria* (~92%) were predominant at 6 d, and *Firmicutes* (~56%) at 4 weeks in the leaves of the uninfected sugar beet genotypes used here (Figure 1A, Table S2). *Beet curly top virus* inoculation significantly altered the relative abundance of bacteria in the leaves in a genotype- (R/S) and stage- (early/late) dependent manner. In general, the resistant Line 4 showed the most variation in bacterial phyla following BCTV infection, both at early (6 dpi) and late (4 wkpi) infection stages (Figure 1A) when compared to its corresponding control and the susceptible Line 19 and the resistant Line 13. Bacterial phyla such as *Mitochondria* and *Rickettsiales* in Line 13 (R) and *Firmicutes*, *Proteobacteria*, *Bacteroidetes*, and *Actinobacteria* increased in Line 4 (R) at 6 dpi. Whereas, an increase in *Clostridia* and *Ruminococcaceae* in Line 13 (R), and *Helicobacteraceae* and *Campylobacterales* in Line 14 (R) at 4 wkpi indicates temporal regulation of the leaf bacterial community in the R lines post BCTV infection. Some of the bacterial phyla/genera detected in our study and their role in disease resistance have also been shown in other systems including plants and animals. In tomato (*Solanum lycopersicum* L.), a lower abundance of *Actinobacteria* and *Firmicutes* in diseased rhizosphere soil (DRS) was highly correlated with disease symptoms caused by *Ralstonia solanacearum* (Smith) Yabuuchi, in comparison to healthy plants that showed higher abundance of these bacteria in the healthy rhizosphere soil (HRS) [25]. Selective disruption of these bacterial communities in HRS increased disease symptoms. Further characterization using individual strains belonging to the two phyla showed their role in activating plant immunity, rather than their antiviral properties, in conferring resistance. In mice, the genus *Akkermansia*, when administered live or pasteurized, showed a reduction in influenza virus titer through its anti-inflammatory and immunoregulatory properties [26].

The variations in the bacterial microbiome observed at the genus level were smaller (vs. phyla level) and highly specific depending upon host genotype and/or BCTV infection (Figure 1B, Table S3). As an example, *Brevibacillus* sp. was highly induced only in the resistant Line 4 at 6 dpi, in comparison to its corresponding control treatment and the susceptible Line 19, suggesting its potential role in BCTV resistance at the early infection stages (Figure 1B). The role of *Brevibacillus* in host plant resistance against viral pathogen has been reported in earlier studies. The antiviral protein BLB8 derived from the *Brevibacillus laterosporus* Laubach strain B8, when co-inoculated, effectively, reduced TMV infection through its direct antiviral properties, along with modulating the expression of host defense-related genes [27]. Tobacco leaf infiltration with pure BLB8 protein, in the absence of the virus, elicited a hypersensitive response (HR) through the production of H_2_O_2_ in a dose-dependent manner. Increased expression of known defense-related genes, namely *PR1*, *PR5a*, *PAL*, and *NPR1*, by three- to five-fold by the BLB8 protein, suggests its role in host defense, priming against the virus and possibly against other pathogens. In another study, the protein elicitor PeBL1, produced by *B. laterosporus*, improved resistance against the insect pest *Myzus persicae* Sulzer in tomato, through the modulation of host plant hormone pathways and altering the physical structures of the leaf surface [28]. Besides *Brevibacillus*, other bacterial genera such as hgcl_clade, *Limnohabitans* were also highly induced at the early (6 dpi) BCTV infection stage in Line 4 (R). Specific roles of these bacterial genera in sugar beet resistance against BCTV are not fully understood at this moment. Future characterization using pure strains of *Brevibacillus* and including other potential candidates detected in this study will allow us to evaluate their relative contribution in BCTV resistance and use as biocontrol agents.

The mode of action by which the microbiome contributes to host plant resistance against biotic and abiotic stress is through the production of metabolites that have direct antimicrobial/antistress properties [13,14]. Some of these metabolites can also modulate host plant stress-related genes/pathways and thereby contribute to resistance. Higher enrichment of the bacterial community associated with metabolites, belonging to the pathways such as polyisoprenoid, the super pathway of L-methionine, was observed only in the resistant Line 4 following BCTV infection at the early infection stage (6 dpi; Figure 6). In fact, under field conditions among the two R lines, Line 4 shows greater resistance than Line 13. Higher induction of potentially beneficial bacterial communities at the early infection stages and their potential contribution towards resistance against viruses/biotic stress might partially explain relatively higher BCTV resistance in Line 4. Both polyisoprenoid and methionine metabolism have been reported to play a critical role in plant resistance against viruses and other pathogens. Resistant tobacco plants, when inoculated with *Tobacco mosaic virus* (TMV) or *Pseudomonas syringae* pv. tabaci, showed significant accumulation of polyisoprenoid alcohols, solanesol (7 to 8-fold) and another family of polyprenols (from 14 to 18 isoprene units with 16 dominating) in the leaves, which increased by seven- to eight-fold and two- to 2.5-fold at 7 dpi [29]. The susceptible tobacco plants on the other hand did not show any increase in these compounds post pathogen inoculation, suggesting the role of polyisoprenoids in resistance against virus/biotic stressor. Methionine-related enzymes, and enzymes related to the methionine cycle (MTC) and associated pathways have been shown to play a key role in host plant resistance against viruses, as reviewed in [30]. Potato (*Solanum tuberosum* L.) lines resistant to the potato virus Y exhibited increased accumulation of methionine in the leaves accompanied by increased expression of specific genes associated with MTC, in comparison to the susceptible line [31]. An increase in the bacterial community associated with polyisoprenoids and/or methionine at the early infection stage (no visible symptoms) possibly contributed to the overall increment of these metabolites in the leaves and augmented BCTV resistance in Line 4. At the later infection stage (4 wkpi; with full blown symptoms) enrichment of bacteria associated with amino acid metabolism such as L-methionine, L-threonine, L-lysine (Figure 7B) in Line 13 (R) and tryptophan biosynthesis (Figure 7C) in Line 4 (R), in comparison to the susceptible Line 19 (Figure 7A), might suggest a potential role of these amino acids in sugar beet resistance against BCTV. In fact, amino acid analysis of the leaves of infected plants at early and late infection stages showed higher accumulation of methionine and lysine, respectively, in the leaves of R lines (to be published elsewhere), which are in line with the enrichment of bacteria associated with the metabolism of these amino acids. Many of these amino acids and/or their derivatives such as lysine catabolite pipecolic acid, as reviewed in [32], and the tryptophan-derived alkaloid, NK0238 [33] have been demonstrated as critical regulators of plant systemic-acquired resistance and antiviral resistance, respectively.

Association among bacterial communities revealed a specific pattern of co-occurrence of the bacterial community. *Mitochondria* were lower in the R lines at 6 dpi and *Oxyphotobacteria* were lower in the R lines at 4 wkpi in comparison to the S line. *Akkermansia* were higher in the R lines at 4 wkpi when compared to the S line. The data presented here might indicate a reciprocal relationship between *Akkermansia* and *Oxyphotobacteria*, where higher abundance of the former might provide a selective advantage against the virus in the R lines at later infection stages (Figure 1, Figure 9 and Figure S3). Higher abundance of hgcl_clade and *Limnohabitans* in the resistant Line 4 at the early infection stage (6 dpi) and a positive correlation between the two bacterial genera might also indicate their potential role in resistance against the virus at early infection stage.

## 4. Materials and Methods

### 4.1. Plant Growth Condition, Viral Infection of Sugar Beet Plants, and Sample Collection

BCTV-susceptible (KDH19-17; Line 19) and BCTV-resistant (KDH13; Line 13 and KDH4-9; Line 4) sugar beet plants at the 5–6 leaf-stage and inside cages were infected with viruliferous BLHs (~6–8 BLH/plant) predominantly carrying California/Logan and severe strains of BCTV [24]. The cages were placed inside a growth chamber with the following conditions: 28 °C (day)/21 °C (night), 16 h (day)/8 h (night) photoperiod, and 20% relative humidity. Uninfected plants were used as the control. Following 6 days post inoculation (dpi) with BLHs, newly emerged leaves from both infected and uninfected plants were collected, flash frozen in liquid N, and stored at −80 °C until further processing. Following leaf sample collection, the BLH inoculated plants were sprayed with the insecticide, Admire^®^ Pro (Bayer CropScience LLC, St. Louis, MO, USA), to eliminate any BLHs. The uninfected plants were also similarly treated with Admire^®^ Pro. The plants were then sprayed with water to remove any residual pesticide and moved to the greenhouse. Infected plants were evaluated for BCTV symptoms at 4 wkpi along with uninfected control plants that did not show any disease symptoms. Newly emerged leaves were collected from both infected and uninfected plants at this timepoint, flash frozen in liquid N, and stored at −80 °C. Typically 4 replicates (uninfected control) and 5 replicates (infected) were collected for each treatment, and each replicate was comprised of a pool of two plants.

### 4.2. Genomic DNA Extraction, PCR Amplification, and 16S rRNA Sequencing

Genomic DNA (gDNA) from sugar beet leaf samples were extracted using the Plant/Fungi DNA Isolation Kit (Norgen Biotek Corp, Thorold, ON, Canada), following the manufacturer’s protocol, and stored at −20 °C until further use. The extracted DNA were then used to set up PCRs. The 16S rRNA primer pair, 341F 341F_5′-CCTACGGGNGGCWGCAG-3′ and 805R_5′-GACTACHVGGGTATCTAATCC-3′ [34] was used for PCR amplification of bacterial DNA. The 5′ ends of the primers were tagged with specific barcodes for each sample and universal sequencing primers. Polymerase chain reaction (PCR) was performed in a total reaction volume of 25 μL that contained 25 ng of template DNA per reaction. The PCR conditions included: an initial denaturation at 98 °C for 30 s; 32 cycles of denaturation at 98 °C for 10 s, annealing at 54 °C for 30 s, and extension at 72 °C for 45 s; followed by a final extension step at 72 °C for 10 min. The PCR products were confirmed by running them on a 2% agarose gel electrophoresis. The PCR products were purified using the AMPure XT beads (Beckman Coulter Genomics, Newton, MA, USA) and quantified by Qubit (Invitrogen, Waltham, MA, USA). The amplicon pools were prepared for sequencing and the size and quantity of the amplicon libraries were assessed on an Agilent 2100 Bioanalyzer (Agilent, Santa Clara, CA, USA) and with the Library Quantification Kit for Illumina (Kapa Biosciences, Woburn, MA, USA), respectively. The libraries were sequenced on a NovaSeq PE250 platform with paired-end reads (2 × 250 bp) according to the manufacturer’s recommendations (LC Sciences, Houston, TX, USA).

### 4.3. Data Analysis

The metagenomic reads were processed using the LC Sciences microbiome data analysis pipeline (LC Sciences, Houston, TX, USA). This workflow includes, (i) quality assessment of reads by removing any short sequences (<150 bp) and ambiguous base calls using a maximum expected error threshold of 1.0, (ii) classifying unique sequences after removing sequencing or PCR errors and chimera sequences, (iii) dereplication, whereby combining all identical sequences into unique sequence reads, and (iv) assignment of a zero-radius operational taxonomic unit (zOTU). Taxonomic classification of final zOTUs was performed using BLASTn against the NCBI (www.ncbi.nlm.nih.gov, accessed on 6 June 2021) database and assigning them into taxonomic level.

Paired-end reads obtained through sequencing were assigned to samples based on their unique barcode and truncated by removing the barcode and primer sequence. Paired-end reads were then merged using FLASH. Quality filtering of the raw reads were performed using specific filtering conditions to obtain high-quality clean tags according to the fqtrim (v0.94). Filtering of chimeric sequences were performed by using Vsearch software (v2.3.4). After dereplication using DADA2, feature sequences were obtained. Sequences with ≥97% similarity were assigned to the same operational taxonomic units (OTUs). Representative sequences were chosen for each OTU. Alpha diversity was used to estimate the complexity of species diversity in each sample and were measured by using 5 different indices including Chao1, observed species, goods coverage, Shannon, and Simpson. These indices were calculated using QIIME2 [35]. Beta diversity was calculated by principal coordinate analysis (PCoA) and used to evaluate differences among samples in relation to species complexity. Cluster analysis was performed using QIIME2 (v1.8.0). The graphs/diagrams were drawn using the ‘R’ package (v3.5.2). Blast was used for sequence alignment, and the feature sequences were annotated with SILVA database for each representative sequence.

Linear discriminant analysis effect size (LEfSe) was used to identify differentially abundant families among the treatments to identify potential biomarker/s [36]. The relative abundance of taxonomic features obtained using the greengenes 13_8 database was subsequently used as input for the analysis of LEfSe using the huttenhower server (https://huttenhower.sph.harvard.edu/galaxy/, accessed on 6 June 2021). The default LDA effect size alpha value (*p* = 0.05) and LDA score (2.0) were used to identify significant differences among groups. Functional assignment of microbial communities was obtained using the phylogenetic investigation of communities by reconstruction of unobserved states (PICRUSt) algorithm [37]. Closed reference OTUs were clustered using the greengenes 13_8 database and resulting ‘.biom’ files were used as inputs for PICRUSt (http://huttenhower.sph.harvard.edu/galaxy/, accessed on 6 June 2021). Following normalization of the input OTU table, metagenome functional predictions were performed using the Kyoto encyclopedia of genes and genomes (KEGG) database [38,39] as a reference. The co-occurrence between the bacterial communities were determined using the sparse correlations for compositional data (SparCC) rank correlation coefficients (Python 2.6.1). A random simulation of 100 datasets was created from the original data and pseudo-*p* values were calculated by estimating the number of the 100 datasets that produced the same correlation with the real data [40].

### 4.4. Statistical Analysis

Statistical analysis was performed using the ‘R’ (v3.5.2) package. Statistical significance reported for any analysis is defined as *p* < 0.05.

### 4.5. Data Availability

The raw data resulting from microbiome sequencing (BioProject ID: PRJNA848809) were submitted to the NCBI SRA database.

### 4.6. Evaluation of BCTV Symptoms and RT-PCR Analysis of BCTV-Infected Sugar Beet Plants

Uninfected control and BCTV-inoculated sugar beet plants at 2 dpi, and 4 wkpi were evaluated for BCTV-related symptoms such as leaf curling and thickening, and vein swelling. Plants representing specific treatments were photographed. Viral load in the infected plant samples was assessed through RT-PCR. Furthermore, cDNA was produced using the iScriptTM gDNA Clear cDNA Synthesis Kit (Bio-Rad, Hercules, CA, USA) following the manufacturer’s protocol. The thermocycler conditions included the pre-incubation step at 98 °C for 30 s, 33 cycles (6 dpi samples)/22 cycles (4 wkpi samples) of 98 °C for 5 s (enzyme activation), 60 °C for 10 s (primer annealing), 72 °C for 3 s (elongation), followed by a final elongation step at 72 °C for 5 min. Equal amount of template cDNA was added for PCR amplification of each sample. Equal volumes of the PCR products were run in a 2% agarose gel. The primers used for BCTV capsid protein gene (*V1*) amplification were, qCP_F-5′-TCCCAGAAAAGAAAGGTGAATCCT-3′ and qCP_R-5′-CCATTGGTATTTCCTCGAAGTCGT-3′. The *V1* gene primers were designed in such a way that they were universal for all BCTV strains found to infect sugar beet. The sugar beet house-keeping gene [*glutamine synthetase* (*GNL2*; EU558132.1)] primers were qBv_F-5′-CCTTCAGGGTGTCGCCAAT-3′ and qBv_F-5′-TTGCCTTCCTTCTCCGTATCA-3′ [24].

## 5. Conclusions

**Figure 10:**
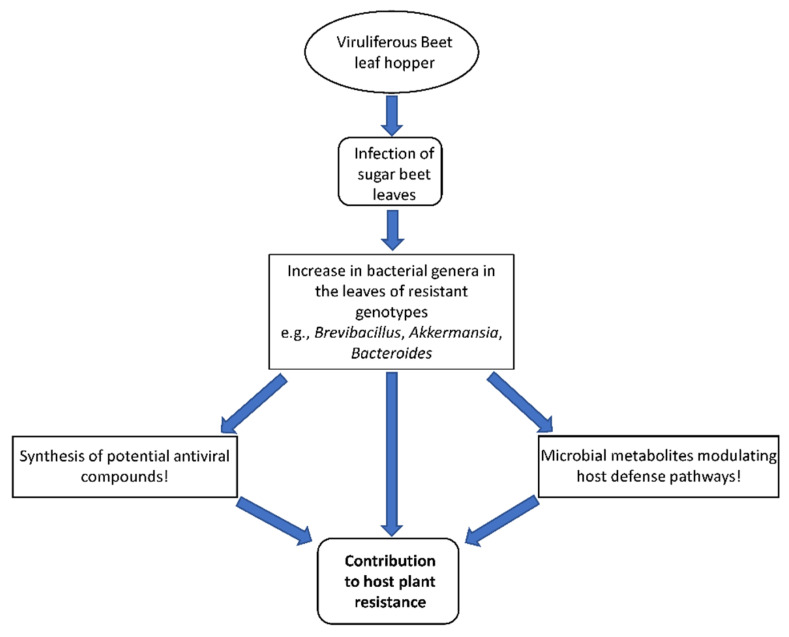
Proposed role of leaf-bacterial-microbiome-mediated sugar beet resistance against *beet curly top virus* in the virus-resistant genotypes.

In this study we have demonstrated the differential responses of the sugar beet leaf bacteriome against BCTV infection during early and late infection stages. Using BCTV-resistant and BCTV-susceptible genotypes, we have identified putative bacterial markers such as *Brevibacillus* and *Akkermansia* that could potentially be associated with virus resistance at early and late BCTV infection stages, respectively (Figure 10). Future characterization using pure strains of candidate bacteria and delineating their role in resistance against BCTV will help in prioritizing their use as potential biocontrol agents, either through seed treatment or foliar spraying under field conditions.

## Data Availability

The authors declare the availability of data and materials upon request.

## Supplementary Materials

The following supporting information can be downloaded at: https://www.mdpi.com/article/10.3390/ijms23158073/s1.
