# Supplementary material for: Leaf Bacteriome in Sugar Beet Shows Differential Response against Beet curly top virus during Resistant and Susceptible Interactions
Source: Int J Mol Sci. 2022 Jul 22;23(15):8073. doi: 10.3390/ijms23158073 (PMC9331236; doi:10.3390/ijms23158073)
Supplement: Supplementary file 1 [file ijms-23-08073-s001.zip › Figures S1-S3.pdf]

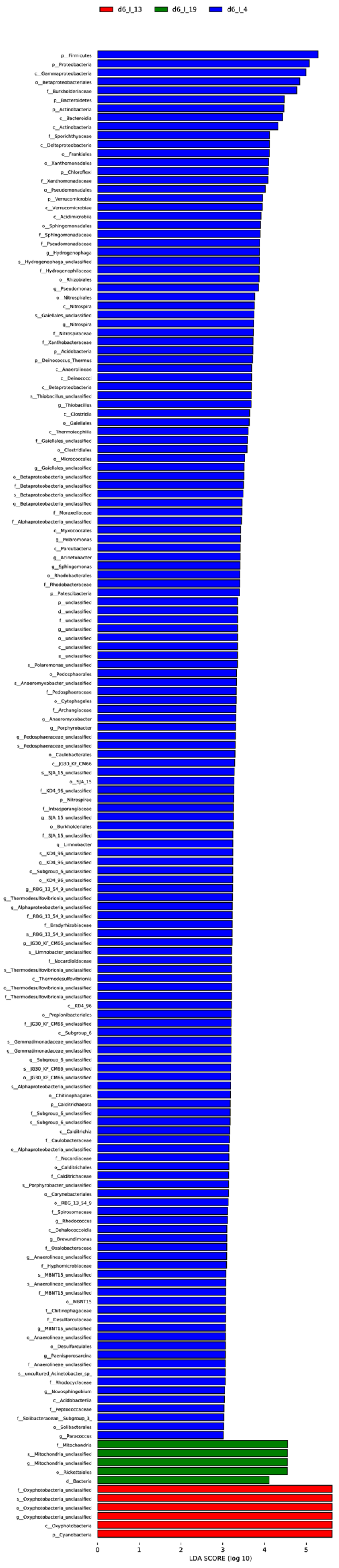

Figure S1. Linear discriminant analysis Effect Size (LEfSe) analysis of sugar beet genotypes at 6 days (d6) post inoculation. Taxonomic abundance of potential biomarkers of *Beet curly top virus* in the susceptible (S) and resistant (R) sugar beet genotypes. 19, S genotype; 13 and 4, R genotypes; I = BCTV-infected.

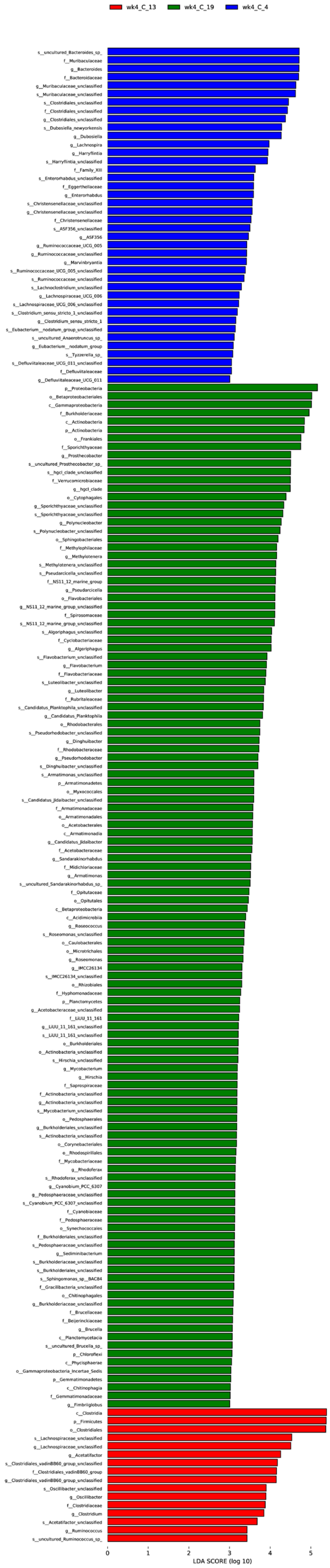

Figure S2A. Linear discriminant analysis Effect Size (LEfSe) analysis of sugar beet genotypes at 4 weeks (wk4). Taxonomic abundance of potential biomarkers in the control of BCTV susceptible and resistant sugar beet genotypes. 19, susceptible genotype; 13 and 4, resistant genotypes; C = uninfected control, and I = BCTV-infected.

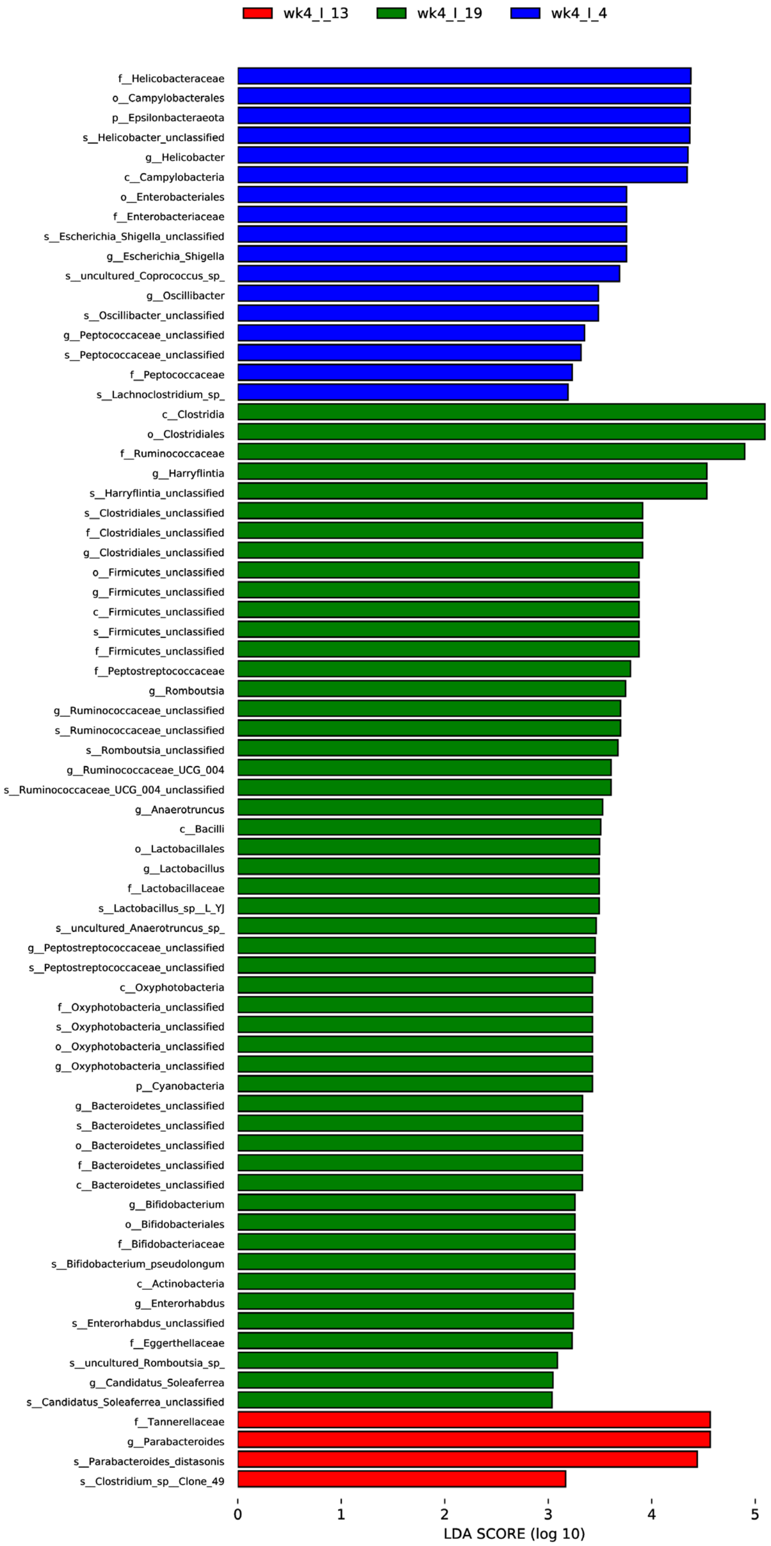

Figure S2B. Linear discriminant analysis Effect Size (LEfSe) analysis of sugar beet genotypes at 4 weeks (wk4). Taxonomic abundance of potential biomarkers of *Beet curly top virus* in the BCTV infected susceptible and resistant sugar beet genotypes. 19, susceptible genotype; 13 and 4, resistant genotypes; C = uninfected control, and I = BCTV-infected.

4 wkpi

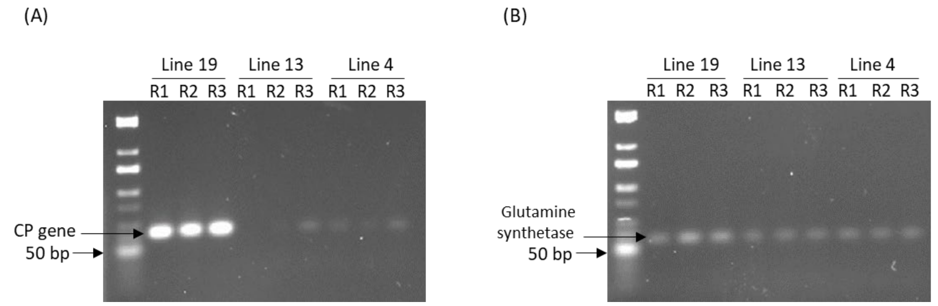

Figure S3. RT-PCR analysis of viral load in the infected leaves of BCTV susceptible (Line 19) and resistant (Line 13, Line 4) sugar beet genotypes at 4 wkpi. (A) Expression of BCTV coat protein (CP) gene, *V1*; (B) expression of sugar beet housekeeping gene, *glutamine synthetase*, in the same samples under the same thermocycling conditions (22 cycles). NEB TriDye™ Ultra Low Range DNA Ladder was used as a marker.
