# Supplementary material for: Leaf Bacteriome in Sugar Beet Shows Differential Response against Beet curly top virus during Resistant and Susceptible Interactions
Source: Int J Mol Sci. 2022 Jul 22;23(15):8073. doi: 10.3390/ijms23158073 (PMC9331236; doi:10.3390/ijms23158073)
Supplement: Supplementary file 1 [file ijms-23-08073-s001.zip › Table S1-S3.pdf]

Table S1. Overview of reads for different samples. C = control, I = infected, and M = million.

Sample	Raw_Tags	Raw_Bases	Valid_Tags	Valid_Bases	Valid%	Q20%	Q30%	GC%
d6_C_19_1	85335	42.67M	71787	29.00M	84.12	93.87	84.47	56.28
d6_C_19_2	82013	41.01M	73960	29.89M	90.18	96.55	90.54	55.93
d6_C_19_3	80373	40.19M	74759	30.20M	93.02	96.40	89.99	56.03
d6_C_19_4	82705	41.35M	73093	29.53M	88.38	96.04	89.15	56.06
d6_C_13_1	82750	41.38M	72896	29.45M	88.09	95.68	88.41	56.07
d6_C_13_2	86736	43.37M	79028	31.93M	91.11	95.81	88.81	56.06
d6_C_13_3	80795	40.40M	67913	27.44M	84.06	95.34	87.65	56.12
d6_C_13_4	85393	42.70M	67643	27.33M	79.21	92.68	82.16	56.41
d6_C_4_1	78520	39.26M	64093	25.89M	81.63	92.80	82.33	56.45
d6_C_4_2	86203	43.10M	78125	31.56M	90.63	96.26	89.67	56.01
d6_C_4_3	87012	43.51M	82847	33.52M	95.21	96.78	91.02	55.97
d6_C_4_4	81990	40.99M	71330	28.82M	87.00	95.16	86.91	56.13
d6_I_19_1	85972	42.99M	78511	31.72M	91.32	96.17	89.59	56.01
d6_I_19_2	86788	43.39M	80274	32.43M	92.49	95.75	88.67	56.07
d6_I_19_3	81295	40.65M	71068	28.71M	87.42	95.76	88.66	56.09
d6_I_19_4	80583	40.29M	67658	27.34M	83.96	94.21	85.14	56.21
d6_I_19_5	83900	41.95M	72272	29.20M	86.14	94.24	85.21	56.25
d6_I_13_1	85539	42.77M	77144	31.17M	90.19	96.46	90.33	56.03
d6_I_13_2	82935	41.47M	77856	31.46M	93.88	96.57	90.49	56.03
d6_I_13_3	80790	40.40M	72139	29.14M	89.29	96.42	90.21	56.04
d6_I_13_4	86457	43.23M	77757	31.41M	89.94	95.93	89.03	56.08
d6_I_13_5	78706	39.35M	70300	28.40M	89.32	95.61	88.33	56.16
d6_I_4_1	83258	41.63M	71312	28.81M	85.65	95.64	88.45	56.10
d6_I_4_2	83737	41.87M	66681	27.59M	79.63	92.56	82.32	54.92
d6_I_4_3	84241	42.12M	72031	30.43M	85.51	93.32	83.59	55.20
d6_I_4_4	80036	40.02M	56078	23.61M	70.07	95.73	88.63	54.10
d6_I_4_5	82181	41.09M	62162	26.00M	75.64	95.35	87.61	52.43
wk4_C_19_1	80068	40.03M	57572	24.08M	71.90	95.98	89.03	51.95
wk4_C_19_2	83021	41.51M	60438	25.15M	72.80	95.14	87.08	51.39
wk4_C_19_3	81101	40.55M	63815	26.71M	78.69	94.50	85.75	53.21
wk4_C_19_4	85060	42.53M	62222	25.68M	73.15	94.73	86.22	54.10
wk4_C_13_1	81131	40.57M	58123	24.02M	71.64	93.08	83.06	54.19
wk4_C_13_2	82614	41.31M	64620	26.29M	78.22	92.71	82.25	55.02
wk4_C_13_3	86060	43.03M	68359	27.90M	79.43	96.64	90.52	54.51
wk4_C_13_4	86377	43.19M	73267	29.85M	84.82	96.79	90.82	54.78
wk4_C_4_1	86401	43.20M	66876	27.30M	77.40	96.47	90.13	54.82
wk4_C_4_2	81929	40.96M	63744	26.09M	77.80	95.96	88.80	54.29
wk4_C_4_3	80164	40.08M	64220	26.40M	80.11	95.81	88.54	53.49
wk4_C_4_4	80482	40.24M	62164	25.62M	77.24	95.36	87.57	53.58
wk4_I_19_1	86927	43.46M	64151	26.28M	73.80	93.55	83.88	54.96
wk4_I_19_2	82340	41.17M	64083	26.06M	77.83	95.04	86.83	55.02
wk4_I_19_3	83944	41.97M	63848	26.26M	76.06	96.17	89.34	55.01
wk4_I_19_4	80613	40.31M	64175	26.26M	79.61	95.79	88.23	54.59
wk4_I_19_5	84164	42.08M	65614	26.74M	77.96	96.18	89.38	55.12
wk4_I_13_1	85004	42.50M	68252	27.94M	80.29	96.05	89.01	54.25
wk4_I_13_2	86084	43.04M	70362	28.93M	81.74	94.87	86.35	53.87
wk4_I_13_3	80635	40.32M	59601	24.63M	73.91	95.53	87.97	53.70
wk4_I_13_4	70238	35.12M	57926	23.83M	82.47	94.07	84.83	53.81
wk4_I_13_5	87187	43.59M	69312	28.49M	79.50	94.71	86.09	54.92
wk4_I_4_1	80440	40.22M	62769	25.81M	78.03	96.19	89.43	54.73
wk4_I_4_2	83633	41.82M	69029	28.31M	82.54	96.41	89.91	53.83
wk4_I_4_3	82843	41.42M	66014	27.06M	79.69	96.42	89.96	53.60
wk4_I_4_4	80077	40.04M	66076	27.18M	82.52	96.25	89.55	53.82
wk4_I_4_5	83907	41.95M	67475	27.65M	80.42	95.77	88.51	54.72

Table S2. Top 30 bacterial phyla (%) detected in the leaves of *Beet curly top virus* susceptible (Line 19) and resistance (Line 13 and Line 4) sugar beet lines. C = control, and I = infected.

Phylum	d6_C_19	d6_C_13	d6_C_4	d6_I_19	d6_I_13	d6_I_4	wk4_C_19	wk4_C_13	wk4_C_4	wk4_I_19	wk4_I_13	wk4_I_4
Cyanobacteria	92.460	92.358	93.940	91.98	95.64	19.06	4.10	0.09	0.10	0.06	0.03	0.02
Firmicutes	0.072	0.011	0.019	0.03	0.04	29.66	11.32	58.33	54.27	66.15	51.24	42.96
Bacteroidetes	0.088	0.005	0.004	0.00	0.00	6.86	24.86	21.75	23.94	17.71	26.37	20.47
Verrucomicrobia	0.005	0.003	0.005	0.00	0.00	1.96	13.21	15.05	18.18	12.67	18.42	29.20
Proteobacteria	7.352	7.623	6.022	7.97	4.31	29.00	29.95	3.25	2.44	2.34	3.04	2.73
Actinobacteria	0.021	0.000	0.006	0.01	0.01	5.42	14.50	1.41	0.85	0.80	0.56	0.38
Epsilonbacteraeota	0.000	0.000	0.000	0.00	0.00	0.15	0.01	0.00	0.00	0.00	0.00	4.01
Chloroflexi	0.001	0.000	0.002	0.00	0.00	2.13	0.21	0.00	0.00	0.00	0.00	0.00
unclassified	0.000	0.000	0.000	0.00	0.00	0.43	0.06	0.12	0.22	0.25	0.32	0.23
Acidobacteria	0.000	0.000	0.000	0.00	0.00	1.11	0.12	0.00	0.00	0.00	0.00	0.00
Gemmatimonadetes	0.000	0.000	0.002	0.00	0.00	0.62	0.18	0.00	0.00	0.00	0.00	0.00
Planctomycetes	0.000	0.000	0.000	0.00	0.00	0.42	0.41	0.00	0.00	0.00	0.00	0.00
Armatimonadetes	0.000	0.000	0.000	0.00	0.00	0.01	0.90	0.00	0.00	0.00	0.00	0.00
Spirochaetes	0.000	0.000	0.000	0.00	0.00	0.64	0.00	0.00	0.00	0.00	0.00	0.00
Kiritimatiellaeota	0.000	0.000	0.000	0.00	0.00	0.33	0.00	0.00	0.00	0.00	0.00	0.00
Latescibacteria	0.000	0.000	0.000	0.00	0.00	0.30	0.00	0.00	0.00	0.00	0.00	0.00
Nitrospirae	0.000	0.000	0.000	0.00	0.00	0.27	0.00	0.00	0.00	0.00	0.00	0.00
Calditrichaeota	0.000	0.000	0.000	0.00	0.00	0.24	0.00	0.00	0.00	0.00	0.00	0.00
Patescibacteria	0.000	0.000	0.000	0.00	0.00	0.14	0.10	0.00	0.00	0.00	0.00	0.00
Rokubacteria	0.000	0.000	0.000	0.00	0.00	0.21	0.01	0.00	0.00	0.00	0.00	0.00
Tenericutes	0.000	0.000	0.000	0.00	0.00	0.16	0.00	0.00	0.00	0.00	0.01	0.00
Lentisphaerae	0.000	0.000	0.000	0.00	0.00	0.16	0.01	0.00	0.00	0.00	0.00	0.00
Fusobacteria	0.000	0.000	0.000	0.00	0.00	0.14	0.00	0.00	0.00	0.00	0.00	0.00
BRC1	0.000	0.000	0.000	0.00	0.00	0.12	0.00	0.00	0.00	0.00	0.00	0.00
Chlamydiae	0.000	0.000	0.000	0.00	0.00	0.08	0.00	0.00	0.00	0.00	0.00	0.00
Omnitrophicaeota	0.000	0.000	0.000	0.00	0.00	0.04	0.01	0.00	0.00	0.00	0.00	0.00
Chlorobi	0.000	0.000	0.000	0.00	0.00	0.04	0.00	0.00	0.00	0.00	0.00	0.00
GAL15	0.000	0.000	0.000	0.00	0.00	0.04	0.00	0.00	0.00	0.00	0.00	0.00
Cloacimonetes	0.000	0.000	0.000	0.00	0.00	0.04	0.00	0.00	0.00	0.00	0.00	0.00
WS2	0.000	0.000	0.000	0.00	0.00	0.04	0.00	0.00	0.00	0.00	0.00	0.00

Table S3. Top 30 bacterial genera (%) detected in the leaves of *Beet curly top virus* susceptible (Line 19) and resistance (Line 13 and Line 4) sugar beet lines. C = control, and I = infected.

Genus	d6_C_19	d6_C_13	d6_C_4	d6_I_19	d6_I_13	d6_I_4	wk4_C_19	wk4_C_13	wk4_C_4	wk4_I_19	wk4_I_13	wk4_I_4
Oxyphotobacteria_unclassified	92.46	92.36	93.94	91.98	95.64	18.90	3.62	0.09	0.10	0.06	0.03	0.02
Akkermansia	0.00	0.00	0.01	0.00	0.00	0.01	3.28	15.05	18.18	12.67	18.42	29.20
Muribaculaceae_unclassified	0.00	0.00	0.00	0.00	0.00	0.05	1.34	9.13	10.00	10.12	8.88	10.05
Bacteroides	0.00	0.00	0.00	0.00	0.00	0.00	0.82	4.94	10.19	4.96	8.28	6.96
Ruminococcaceae_UCG-013	0.00	0.00	0.00	0.00	0.00	0.00	3.43	4.94	4.32	10.12	5.92	4.76
Lachnospiraceae_NK4A136_group	0.00	0.00	0.00	0.00	0.00	0.00	1.15	8.31	3.83	7.91	5.55	6.30
Mitochondria_unclassified	7.20	7.60	5.20	7.95	4.30	1.49	0.00	0.00	0.00	0.00	0.00	0.00
Brevibacillus	0.00	0.00	0.00	0.03	0.04	28.22	0.05	0.00	0.00	0.01	0.00	0.00
Lachnospiraceae_unclassified	0.00	0.00	0.00	0.00	0.00	0.06	0.79	7.33	4.75	5.59	4.18	4.01
Dubosiella	0.00	0.00	0.00	0.00	0.00	0.00	0.93	1.35	4.57	4.58	7.29	4.37
Eisenbergiella	0.00	0.00	0.00	0.00	0.00	0.01	1.07	4.30	2.46	5.09	2.32	2.97
Clostridiales_unclassified	0.00	0.00	0.00	0.00	0.00	0.00	0.20	1.98	4.99	3.87	2.34	2.38
Parabacteroides	0.00	0.00	0.00	0.00	0.00	0.00	4.64	4.15	0.00	0.00	7.29	0.00
Limnohabitans	0.00	0.00	0.00	0.00	0.00	3.89	11.47	0.00	0.01	0.00	0.00	0.00
Parasutterella	0.01	0.00	0.00	0.00	0.00	0.00	1.69	3.24	2.31	2.22	2.27	1.44
Acetatifactor	0.00	0.00	0.00	0.00	0.00	0.00	0.06	3.77	3.56	2.29	1.80	1.52
Clostridiales_Family_XIV._Incertae_Sedis_unclassified	0.00	0.00	0.00	0.00	0.00	0.00	0.33	3.14	2.31	1.27	1.97	1.68
A2	0.00	0.00	0.00	0.00	0.00	0.00	0.23	3.13	1.47	1.25	2.99	1.41
Harryflintia	0.00	0.00	0.00	0.00	0.00	0.00	0.08	1.01	1.80	6.19	0.61	0.00
Clostridiales_vadinBB60_group_unclassified	0.00	0.00	0.00	0.00	0.00	0.00	0.06	2.98	2.49	0.63	3.05	0.51
Alistipes	0.00	0.00	0.00	0.00	0.00	0.00	0.55	2.40	2.00	1.88	1.89	0.69
Intestinimonas	0.00	0.00	0.00	0.00	0.00	0.00	0.14	1.68	2.00	2.01	1.76	1.26
hgcI_clade	0.00	0.00	0.00	0.00	0.00	1.68	5.82	0.00	0.00	0.00	0.00	0.00
Prosthecobacter	0.00	0.00	0.00	0.00	0.00	0.20	7.32	0.00	0.00	0.00	0.00	0.00
Clostridium	0.00	0.00	0.00	0.00	0.00	0.00	0.23	1.65	1.27	1.12	1.19	0.92
Ruminiclostridium_9	0.00	0.00	0.00	0.00	0.00	0.00	0.12	1.23	1.35	1.26	1.32	0.90
Romboutsia	0.00	0.00	0.00	0.00	0.00	0.00	0.86	1.31	2.49	1.22	0.33	0.06
Oscillibacter	0.00	0.00	0.00	0.00	0.00	0.00	0.04	1.57	0.88	0.61	0.81	1.22
Muribaculum	0.01	0.00	0.00	0.00	0.00	0.00	0.77	1.12	1.72	0.70	0.00	0.68
Firmicutes_unclassified	0.00	0.00	0.00	0.00	0.00	0.03	0.26	0.40	0.61	1.65	1.18	0.26
Others	0.30	0.03	0.84	0.04	0.02	45.45	48.66	9.80	10.33	10.71	8.32	16.41
